# Translation initiation factor IF2 contributes to ribosome assembly and maturation during cold adaptation

**DOI:** 10.1093/nar/gkz188

**Published:** 2019-03-27

**Authors:** Anna Brandi, Lolita Piersimoni, Naser Aliye Feto, Roberto Spurio, Jean-Hervé Alix, Frank Schmidt, Claudio O Gualerzi

**Affiliations:** 1 Laboratory of Genetics, University of Camerino, 62032 Camerino (MC), Italy; 2 Interfaculty Institute for Genetics and Functional Genomics, University Medicine Greifswald, Felix-Hausdorff-Str. 8, 17475 Greifswald, Germany

## Abstract

Cold-stress in *Escherichia coli* induces *de novo* synthesis of translation initiation factors IF1, IF2 and IF3 while ribosome synthesis and assembly slow down. Consequently, the IFs/ribosome stoichiometric ratio increases about 3-fold during the first hours of cold adaptation. The IF1 and IF3 increase plays a role in translation regulation at low temperature (cold-shock-induced translational bias) but so far no specific role could be attributed to the extra copies of IF2. In this work, we show that the extra-copies of IF2 made after cold stress are associated with immature ribosomal subunits together with at least another nine proteins involved in assembly and/or maturation of ribosomal subunits. This finding, coupled with evidence that IF2 is endowed with GTPase-associated chaperone activity that promotes refolding of denatured GFP, and the finding that two cold-sensitive IF2 mutations cause the accumulation of immature ribosomal particles, indicate that IF2 is yet another GTPase protein that participates in ribosome assembly/maturation, especially at low temperatures. Overall, these findings are instrumental in redefining the functional role of IF2, which cannot be regarded as being restricted to its well documented functions in translation initiation of bacterial mRNA.

## INTRODUCTION

After a cold-stress (e.g. a temperature downshift from 37°C to <20°C), mesophilic bacteria like *Escherichia coli* undergo a cold adaptation phase during which gene expression is reprogrammed and a set of cold-shock proteins is synthesized whereas bulk protein synthesis stops (1–8). Transcriptional and post-transcriptional regulations are responsible for these events (8–14). A key role among the post-transcriptional events is played by ‘cold-shock translational bias’ whereby three types of mRNAs have been described, depending upon their translational efficiency at cold-shock temperature. These are cold-shock mRNAs such as *cspA, hns* and *hupB* mRNAs, cold-tolerant mRNAs such as *hupA* mRNA and non-cold-shock mRNAs such as *cspD* mRNA (13). Translational bias is due to both cis-acting and trans-acting elements and ensures selective translation of cold-shock mRNAs and inhibition of non-cold-shock mRNAs (7,12–18). A typical cis-acting element is the peculiar structure of *cspA* mRNA, which assumes different conformations at high (i.e. 37°C) and low (i.e. ≤20°C) temperature, thereby acting as a thermosensor (14,18). The initiation factors IF3 and IF1 and cold-shock protein CspA, whose *de novo* syntheses are stimulated by cold-shock (1,15,19–21) are among the trans-acting elements. Indeed, the increased level of IF1 and IF3 with respect to the ribosomes, which are synthesized and assembled at a highly reduced rate (22), ensures a sufficient supply of dissociated subunits at low temperature and favours the initial steps of translation initiation with cold-shock mRNAs and the rejection of 30S initiation complexes assembled at low temperature with non-cold-shock mRNAs (13,18). CspA and RNase R ensure unhindered translational elongation of cold-shock mRNAs and degradation of overly structured mRNAs (21,23).

In the accompanying article (Brandi *et al.*), it has been shown that cold-shock activates essentially all promoters present in the *nusA-infB* operon (24) and causes a substantial stabilization of the *infB* transcript as well as its increased translational efficiency. As a result, after cold stress also the level of initiation factor IF2 is increased to approximately the same extent as IF1 and IF3. However, so far no role could be attributed to the cold stress-induced IF2 in determining either translational bias or any other activity required for cold acclimation.

In this study, we have investigated a possible function of IF2 during cold adaptation and present several lines of evidence indicating that this factor plays an important role in ribosome assembly and/or maturation in cold-shocked cells, likely by virtue of its protein chaperone activity accompanied by GTP hydrolysis.

## MATERIALS AND METHODS

### Buffers

Buffer A: 10 mM Tris–HCl (pH7.4), 10 mM MgCl_2_, 60 mM NH_4_Cl, 400 mM NaCl, 1 mM DTT; Buffer B: 40 mM Tris–HCl (pH 7.5), 150 mM NaCl, 10% (v/v) glycerol; Buffer C: 50 mM Tris–HCl, (pH 7.5), 0.3 mM EDTA, 1 mM DTT; Buffer D: 50 mM Tris–HCl, (pH 7.5), 25 mM MgCl_2_, 100 mM KCl; Buffer E: 40 mM Tris–HCl, (pH 7.8), 50 mM NaCl, 20 mM KCl, 20 mM MgCl_2_, 5 mM β-mercaptoethanol and 10% glycerol.

### Bacterial strains

*Escherichia coli* BL21*infB::kanR*, whose chromosomal copy of *infB* is inactivated by insertion of a kanamycin cassette but bearing a copy of wt *infB* on a plasmid containing a thermo-sensitive origin of duplication (25), was transformed with P_BAD_-*infB* encoding the natural long form of IF2 (IF2α) or P_BAD_-*infB*ΔN, encoding a protein lacking the entire N-terminal domain (NTD) of the factor (Supplementary Figure S1). The transformations yielded *E. coli* BL21*infB::KanR* P_BAD_-*infB* or *E. coli* BL21*infB::KanR* P_BAD_-*infB*ΔN in which the thermo-sensitive plasmid carrying wt *infB* was eliminated by exposure to the non-permissive temperature (25). For the sake of simplicity these cells will be referred to as *E. coli* IF2α and *E. coli* IF2ΔN, respectively. It should be noted here that whereas full expression of the *infB* genes cloned in these plasmids requires the induction with arabinose, the P_BAD_ promoter is leaky enough so as to ensure the production of a sufficient amount of factor also in the absence of the inducer.

*Escherichia coli* BL21*infB::KanR* pGEX*infBE571K*, expressing an IF2α molecule bearing a single amino acid substitution (E571K) which completely inactivates the GTPase activity (26) was also used in this study and for simplicity will be referred to as *E. coli* IF2αΔGTPase.

A scheme showing the structures of *E. coli* IF2α, IF2β and IF2ΔN, as well as the location of the E571K substitution which inactivates the GTPase activity of the factor is presented in Supplementary Figure S1.

### Protein overproduction and purification

For protein renaturation experiments a GFP mutant (i.e. GFP S30R) was used. Overproduction and purification of the green fluorescent protein GFP S30R mutant was performed as described (27). Initiation factors IF2α, IF2β (28), IF2ΔN (29) and IF2 E571K (26) were obtained and purified as described. Elongation factor EF-G was a kind gift of Prof. A. Dahlberg (Brown University, Providence, RI, USA).

### Cell labeling with ^3^H uridine and ^15^N for ribosome analysis

*Escherichia coli* IF2α, *E. coli* IF2ΔN and *E. coli* IF2αΔGTPase strains were grown at 37°C in M9 medium supplemented with Casamino acids. Upon reaching *A*_600_ = 0.35 each cell culture was divided into two 30 ml aliquots. The first aliquot was labeled for 20 min at 37°C by addition of 50 μl of 28 μM uridine containing 36 Ci/mM 5-[^3^H] uridine (30) while the second aliquot was transferred to 10°C and 1 h after the temperature downshift was exposed for 1 h to the same amount of radioactive uridine.

For ^14^N/^15^N mass spectrometry (MS) analysis, two identical cultures were grown at 37°C in M9 minimal medium (2 mM MgSO_4_, 22 mM KH_2_PO_4_, 20 mM Na_2_HPO_4_, 8.5 mM NaCl, 0.5 mg/ml NH_4_Cl, 5 mg/ml glucose, 50 μg/ml thiamine) containing either heavy ^15^NH_4_Cl or light ^14^NH_4_Cl until reaching *A*_600_ = 0.6. The heavy labeled reference cells were then harvested and divided into 18 aliquots. A sample (25 ml) of the cells exposed to ^14^N was collected before transfer to 10°C (time = 0) and subsequent samples were collected at different points in time after the temperature downshift. For each nano-LC–MS/MS analysis, identical numbers of cells were taken from the light and heavy labeled cultures and then mixed. Preliminarily, the number of cells in each sample was determined by viable counting. For this an aliquot (1 ml) of the culture was taken at each time point and subjected to four serial dilutions (1:20; 1:20; 1:20; 1:10); 50 μl from the last dilution were spread on a plate. After incubation at 37°C overnight the colonies were counted.

For both preparations, cells were collected by centrifugation at 5 K rpm for 5 min (Sorvall, GSA rotor), washed with ice‐cold physiological saline solution (0.9% NaCl) and pelleted by centrifugation at 10 K rpm for 2 min in an Eppendorf tube and kept frozen until further analysis.

### Sucrose gradient analysis of ribosomal particles

Extracts of the cells exposed to ^3^H uridine at 37°C or 10°C were loaded onto 14 ml 10–30% sucrose gradients prepared in buffer A. After 16 h centrifugation at 4°C at 27 K rpm in a TST41SW rotor (Kontron) the gradients were fractionated and the *A*_260_ and the radioactivity of each fraction measured essentially as described (30).

Cells grown in the presence of heavy ^15^NH_4_Cl and light ^14^NH_4_Cl were resuspended in ice-cold buffer A and mixed in the right proportion based on the viable counts, disrupted by sonication and subjected to 20 min centrifugation at 14 K rpm at 4°C in a table-top Eppendorf centrifuge to separate cell debris from the crude extract. This was then loaded onto 10%-30% sucrose gradients prepared in the above buffer and centrifuged at 24 K rpm for 16 h at 4°C in a SW41 rotor (Beckman).

### Mass spectrometry (MS) analyses

**Figure 1.**
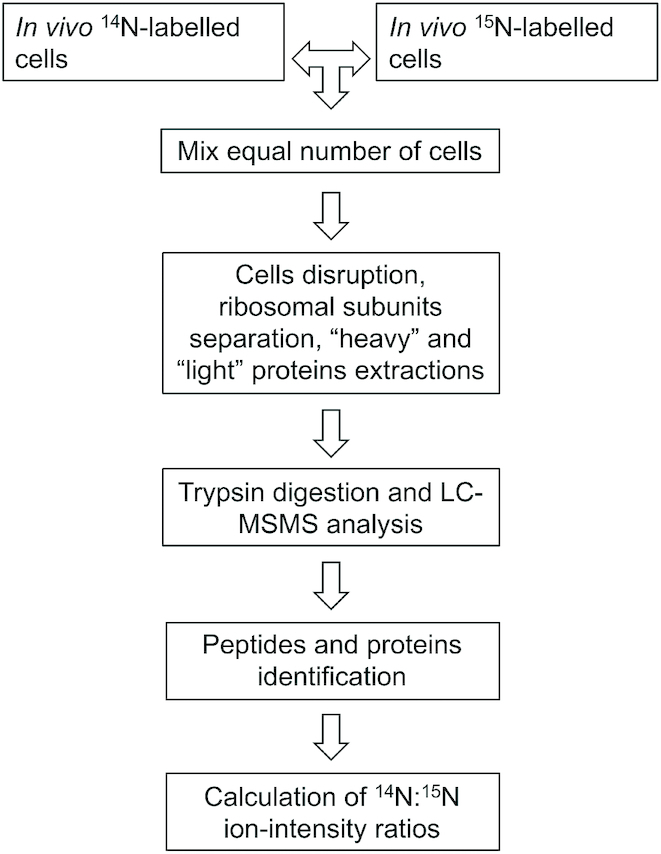
Flow chart illustrating the experimental strategy used for the mass spectrometry analyses. Two cell populations were grown in parallel in M9 culture media, identical except for the presence of ^15^NH_4_Cl in one case and of ^14^NH_4_Cl in the other. This results in the synthesis of ‘heavy’ and ‘light’ proteins, respectively. The culture to be evaluated (light) and the reference culture (heavy) were mixed together just before each step of sample preparation, thus reducing the experimental error that would distort the quantitative result. The proteins were digested and prepared for the LC–MS/MS analysis, and the data processed using software for quantitative proteomics that can handle metabolic labeling (see Materials and Methods). The MS/MS spectra were used to identify the peptides, the software algorithm for quantitative proteomics extracted individual MS1 isotopes and calculated peptide AUC ratios for each peptide pair. The weighted average of all peptide ratios was calculated for each protein as a value for the protein ratio.

The overall strategy used in these experiments (31) is indicated in the scheme presented in Figure 1 and described in more detail in the Results section. After determining their concentration by Bradford assay, 2 μg of total proteins in NH_4_HCO_3_ (20 mM) were digested with 80 ng modified porcine trypsin at 37°C overnight. The reaction was stopped by adding acetic acid to a final concentration of 1%. The samples were purified by μC18 ZipTip (Millipore, Billerica, MA, USA). After equilibration and binding of peptides as indicated by the manufacturer, peptides were washed with 0.1% acetic acid and eluted first with 5 × 5 μl 50% acetonitrile (ACN), then with 5 × 5 μl 80% ACN in 0.1% acetic acid. The total eluate was concentrated in a vacuum centrifuge (Konzentrator 5301) to a final volume of 5μl. Subsequently, 7 μl of 0.1% CH_3_COOH/2% ACN were added to the samples. The peptide solutions were analyzed using a Proxeon Easy nLC (Proxeon Biosystems A/S, Denmark) connected to an LTQ-Orbitrap XL (Thermo Electron Corporation, Bremen, Germany) equipped with a nano-ESI source. The acquisition was performed for 300 minutes. In detail, the peptides were separated using an analytical column Acclaim PepMap 100 (C18, particle size 3 μm, 100 Å, manufactured by LC-Packings, Dionex, USA) of 15-cm bed length. The peptides were enriched on a pre-column, Biosphere C18 (ID 100μM, particle size 5 μm, length 20 mm, pore size 120 Å, by NanoSeparations, Netherlands). The peptides were eluted at a flow rate of 300 nl/min using a non-linear solvent gradient of buffer F (0.1% acetic acid and 2% ACN) and buffer G (0.1% acetic acid in ACN), from 0% G to 60% G in 290 min at a flow rate of 300 nl/min. The eluted peptide mixture was infused into mass spectrometer by nano-ESI source. The MS was operated in positive ion mode and data-dependent mode to automatically switch between Orbitrap-MS and MS/MS acquisition. Survey full scan MS spectra (from m/z 300 to 2000) were acquired in the Orbitrap at 60 000 resolution. The method used allowed sequential isolation of maximum five most intense ions depending on signal intensity; they were subjected to collision-induced fragmentation (CID) with an isolation width of 2 Da. Target ions already selected for MS/MS were dynamically excluded for 30 s. General MS conditions were electrospray voltage, 1.7 kV, no sheath and auxiliary gas flow, capillary temperature of 200°C. Ion selection threshold was 1000 counts for MS/MS, activation time of 30 ms and activation energy of 35% normalized were also applied for MS/MS. A scan range was automatically selected depending on the m/z value measured for the precursor ion. Only doubly and triply charged ions were triggered for tandem MS analysis. Differential analysis of label‐free MS data was performed using Sorcerer™-SEQUEST^®^ (version 3.5, Sage‐N Research, Bioworks, Milpitas, CA, USA); briefly, peptide identification was performed by the Sorcerer platform using the integrated SEQUEST algorithm (10 ppm precursor tolerance and 0.5 Da for MS/MS fragment ions mass tolerance as parameters) and the quantification was done with the ^14^N/^15^N option in Xpress as a part of the integrated Trans-Proteomic Pipeline (TPP) in Sorcerer. After identification of the light and heavy peptides, the MS1 data with a precursor tolerance of 0.2 Da were extracted from the mzXML files, and the AUC (Area Under the Curve) data of each peptide per single protein was taken to calculate each protein ratio based on the geometric mean of the constituent peptides.

Subsequently, the ratios of each single protein was normalized to the average level of all ribosomal proteins of the small subunit at each time point and then taking each ratio before the stress as being = 1.

### Spontaneous and chaperone-assisted refolding of denatured GFP

Heat denatured wt GFP fails to renature under all conditions tested. Therefore, to study spontaneous and chaperone-assisted refolding of denatured GFP, we used a modified GFP protein bearing an S30R substitution which affects both reversibility of denaturation and refolding. This reporter protein was subjected to either heat or acid denaturation. Heat denaturation was carried out at 95°C for 8 min in Buffer B, essentially as described (32) whereas acid denaturation was carried out by mixing a 5 μM GFP solution in Buffer C with an equal volume of 125 mM HCl, essentially as described (33). Chaperone-assisted GFP refolding was studied by following fluorescence emission of this protein in real time using a qPCR (Mx3000P Stratagene) machine equipped with a FAM filter set at 492 nm (excitation) and 516 nm (emission) wavelengths, which were within the range of excitation and emission wavelengths of the GFP used in this study. Several tests were carried out to find the linear range of the qPCR instrument, the range at which the fluorescent signal is linear with the quantity of fluorescent GFP. The fluorescence was found to increase up to approximately ≥20 pmol of fully folded GFP, the signal being linear between 1.5 and 12 pmol of protein (Supplementary Figure S2). Thus, to monitor spontaneous and chaperone-assisted refolding of denatured GFP, the samples containing 3.75 pmol of GFP (final concentration 0.075 μM) in 50 μl of Buffer D (for acid-denatured GFP) or Buffer E (for heat denatured GFP) supplemented with the indicated amounts of proteins to be tested for chaperone activity (IF2α, IF2β, IF2ΔN, IF2αΔGTPase, EF-G, DnaK, DnaJ, GrpE) were placed into qPCR tubes (Axygen, USA); the temperature was set at 25°C or at 17°C to mimic the condition occurring during cold-adaptation *in vivo* without removing the samples from the qPCR tubes. Subsequently, the fluorescence data were collected every 2–3 min.

## RESULTS

### The level of ribosome-associated IF2 increases during cold adaptation

To find a role for IF2 during cold acclimation, experiments were designed to determine the location of the extra copies of this factor produced after the cold stress.

**Figure 2.**
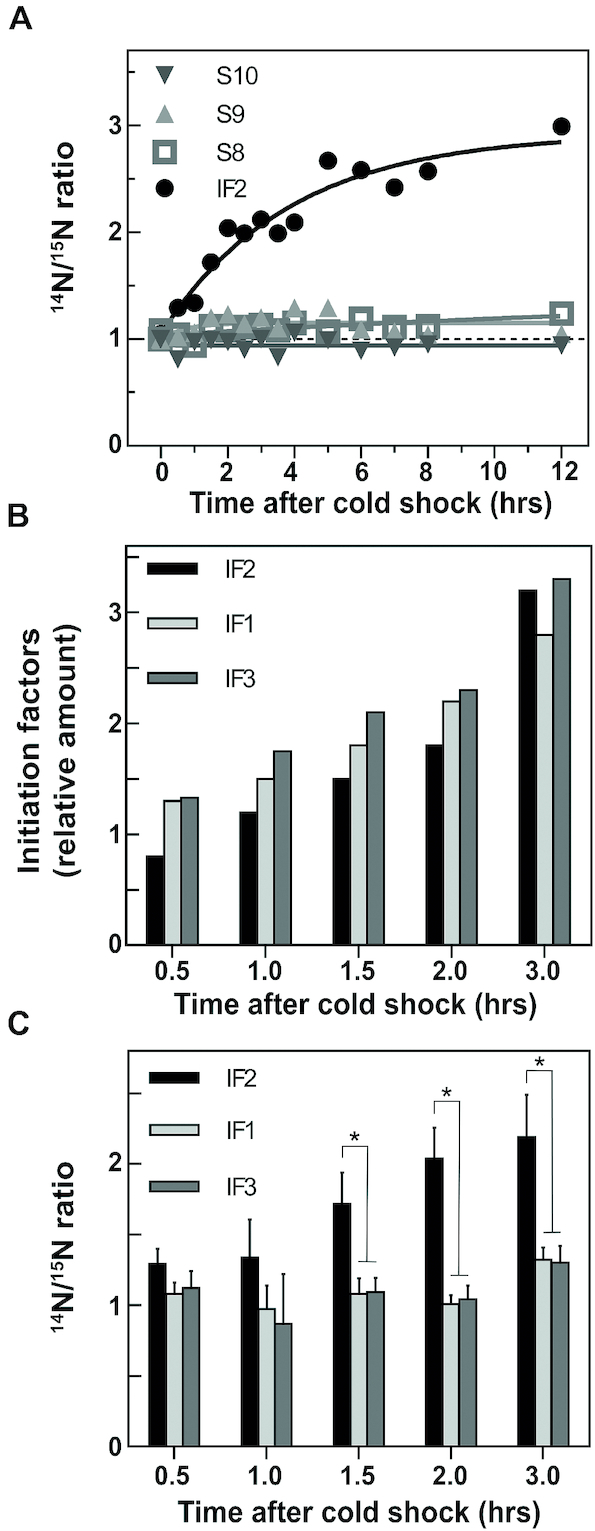
Level of initiation factors and ribosomal proteins associated with the ribosomal subunits during cold acclimation. (**A**) Variation, as a function of the time elapsed after cold stress (37°C →10°C), of the levels of IF2 (•), S8 (□), S9 (▵) and S10 (▿) associated with the peak of the 30S ribosomal subunits resolved by sucrose density gradient centrifugation. The relative protein levels after the cold stress were determined from the ^14^N/^15^N ratios taking the ratio recorded just before the stress as = 1; (**B**) variation of the relative cellular levels of IF1 (light grey bars), IF2 (black bars) and IF3 (dark grey bars) with respect to the amount of ribosomes as a function of the hours elapsed after cold shock. The protein levels, determined by western blotting are normalized taking the levels detected before the stress as = 1. This experiment is described in detail in (13) from which the panel is taken after some modifications and is shown here only to emphasize the difference between total level of free IFs in the cell and their levels in association with the ribosomes; (**C**) variation as a function of the hours elapsed after cold shock of the^14^N/^15^N ratios determined for IF1 (light grey bars), IF2 (black bars) and IF3 (dark gray bars) found in association with the 30S subunits. The levels detected before the stress are taken as being = 1. The standard deviation for each protein was measured using the respective pool of peptide ratio measurements. *P ≤ 0.001 (ANOVA test, IF2 versus IF1 or IF3, was performed using average ratio, standard deviation and number of identified peptides of each protein).

Thus, two cell cultures were grown in parallel in M9 media, identical except for the presence of ^15^NH_4_Cl in one case and of ^14^NH_4_Cl in the other resulting in the synthesis of ‘heavy’ and ‘light’ proteins, respectively. Both reference and test cells were grown at 37°C till they reached *A*_600_ = 0.6. The ‘light’ culture, exposed to ^14^N, was then subjected to a 37°C to 10°C cold stress after which aliquots were withdrawn at different times during the subsequent cold acclimation. The ‘heavy’ culture, exposed to ^15^N, was divided into as many aliquots as the number of samples of ‘light’ cells taken during cold-adaptation. For each nano-LC–MS/MS experiment, an identical number of cells from the ‘heavy’ and ‘light’ cultures, as assessed from viable counts determination, were mixed just before each step of sample preparation, thus reducing the experimental error that would distort the quantitative result. The cells were ruptured by sonication to yield extracts which were subjected to sucrose density gradient centrifugation to determine if any change in the stoichiometry of the proteins associated with the ribosomal subunits had occurred during cold-acclimation. For this purpose, the fractions corresponding to the peak of the 30S subunits and likely containing also immature precursor particles of both subunits were pooled; the ^14^N/^15^N isotopic ratio was measured for each resulting sample allowing us to quantify the relative amounts of different proteins identified by MS. The ^14^N/^15^N ratio was found to remain constant for all 30S ribosomal proteins for up to 32 h after the cold shock, their ratio ranging from 0.5 to 2.5 which was not considered to be a significant variation with respect to an average ratio of ∼1.35. The isotopic ratios for all ribosomal proteins recorded from the onset of cold stress until after 12 h of cold acclimation, after normalization as indicated in Materials and Methods, are presented in the supplementary materials (Supplementary Figures S3–S6). Unlike the levels of ribosomal proteins and initiation factors IF1 and IF3, it can be seen from the ^14^N/^15^N ratios that, compared to the pre-stress situation, the amount of IF2 molecules associated with the 30S subunit peak increases up to 3-fold during the first 12 h of cold acclimation (Figure 2A) and remains >2-fold higher for up to 32 h after the cold stress. In the same time period the stoichiometry of three 30S ribosomal proteins (S8, S9 and S10) remains constant (Figure 2A). These proteins were selected as being representatives of primary (S8), secondary (S9) and tertiary (S10) proteins in the *in vitro* (34,35) assembly map and of two different hierarchical clusters of the *in vivo* (36) assembly map. Aside from that of IF2, also the ^14^N/^15^N ratio of other proteins sedimenting with the 30S peak was found to increase substantially during cold acclimation; it is noteworthy that most of these proteins are known cold shock proteins involved in ribosome assembly and maturation, such as CsdA (Supplementary Figure S3A), MraW (Supplementary Figure S3B), SrmB (Supplementary Figure S3C), PNPase (Supplementary Figure S4A), RbfA (Supplementary Figure S4B), YibL (Supplementary Figure S4C), RNaseR (Supplementary Figure S5A), LepA (Supplementary Figure S5B), and KsgA (Supplementary Figure S5C). The increased stoichiometry of these proteins during cold-acclimation was found to range from a maximum of 20-fold for CsdA to a minimum of 2.2 for KsgA.

It is noteworthy that whereas the total level of all three initiation factors increases to approximately the same extent with respect to the ribosomes during cold acclimation (Figure 2B), only the extra molecules of IF2 synthesized after cold shock are found in association with the 30S peak (Figure 2C) along with several other proteins involved in ribosome assembly/maturation. The difference between IF2 and the other two initiation factors is statistically significant with the *P* value being <0.01 after 1.5 h cold stress. In turn, these findings indicate that IF1 and IF3 on the one hand and IF2 on the other hand play different roles during cold acclimation.

In addition to the above-mentioned proteins whose stoichiometry on the ribosome increases during cold acclimation and those like all the 30S proteins and IF1 and IF3 whose levels remains constant, there are other proteins (e.g. the methyltransferase RsmE) whose level on the ribosomal subunit decreases substantially after the stress (Supplementary Figure S6).

### Two cold-sensitive IF2 mutants

Several IF2 mutants had previously been constructed in our laboratory. In light of their phenotypes, two of these mutants were investigated in this study. The first (IF2ΔN) bears a deletion of the entire N-terminal domain of the protein (29), as shown in Supplementary Figure S1 The second, which bears a single amino acid substitution (E571K) within the G3 domain of the factor (Supplementary Figure S1) results in a protein completely defective in GTPase activity (26) and is referred to as IF2αΔGTPase.

**Figure 3.**
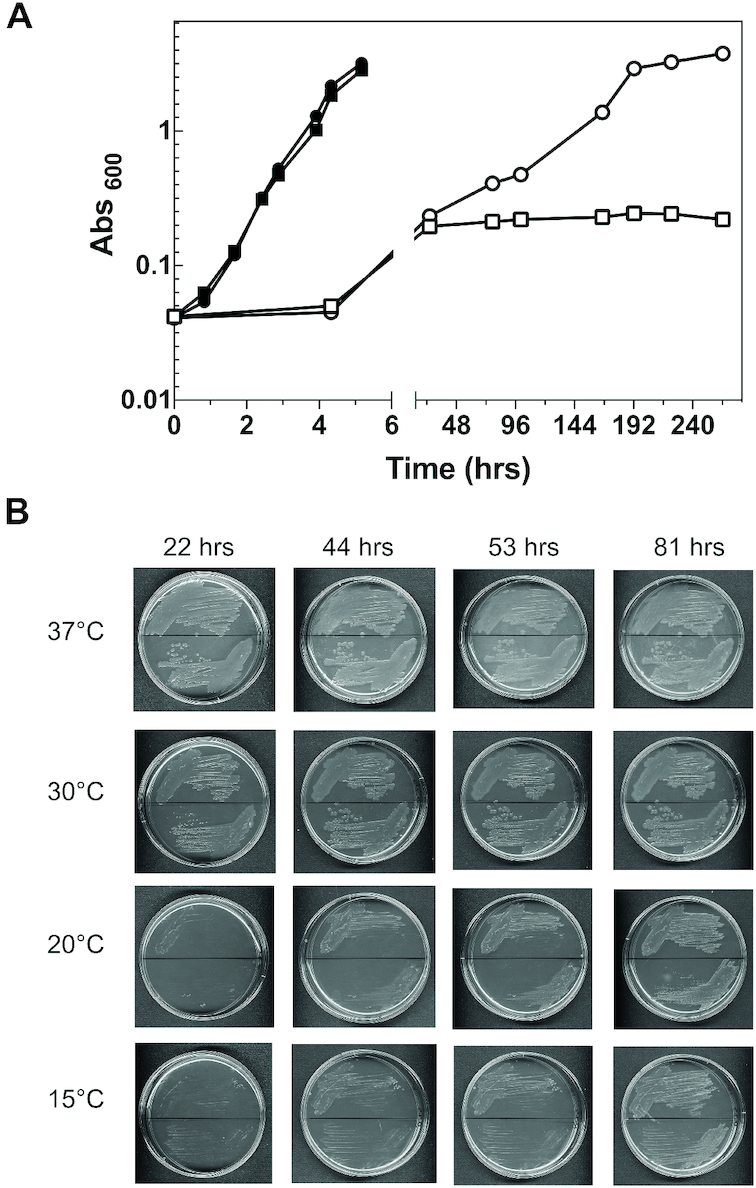
Deletion of the entire NTD of IF2 confers a cold-sensitive phenotype to cells in liquid and solid medium. (**A**) Growth curves at 37°C (•,▪) and 10°C (○,□) in liquid LB medium containing Amp (60 μg/ml), Kan (25 μg/ml) and 0.025% arabinose of *E. coli* cells expressing IF2α (•,○) or IF2ΔN (▪,□); (**B**) growth of *E. coli* cells expressing IF2α (upper half of the plates) or IF2ΔN (lower half of the plates) on LB agar plates containing 0.025% arabinose and the same concentrations of antibiotics indicated above. Growth was for the indicated times at the indicated temperatures. The plates are divided into upper and lower halves by a horizontal line.

**Figure 4.**
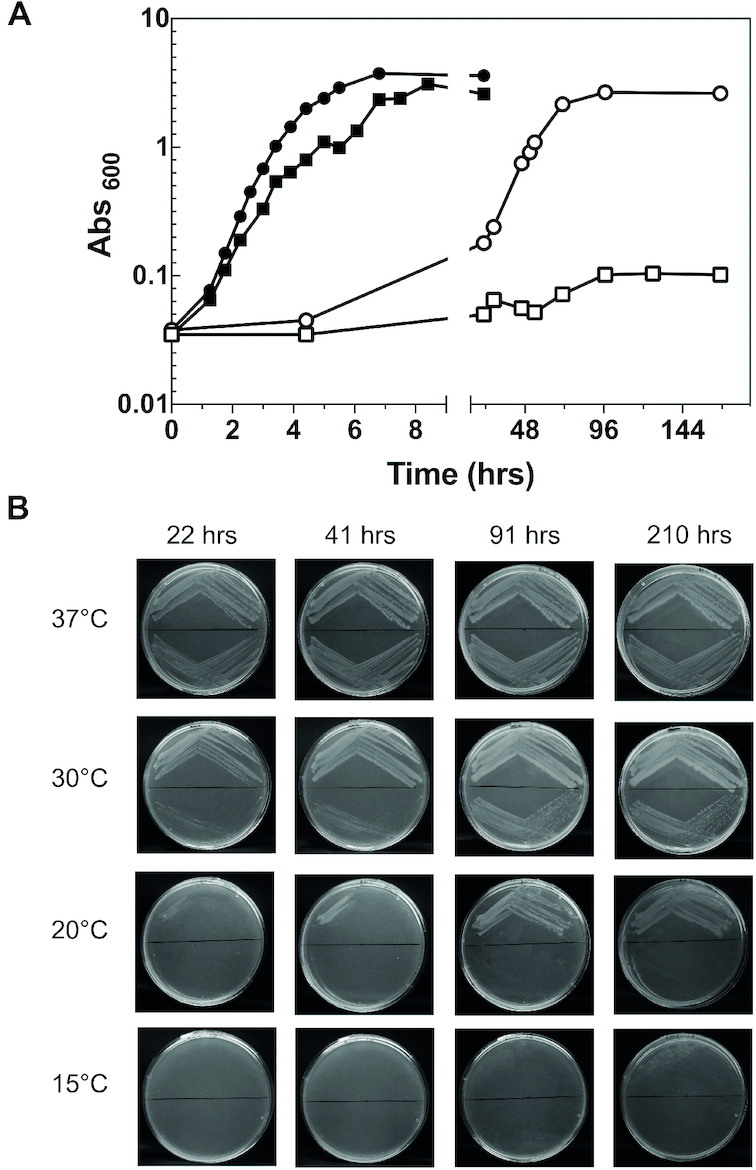
Inactivation of the GTPase activity of IF2 confers a cold-sensitive phenotype to cells in liquid and solid medium. (**A**) Growth curves at 37°C (•,▪) and 15°C (○,□) in liquid LB medium containing Amp (60 μg/ml) and Kan (25 μg/ml) of *E. coli* cells expressing IF2α (•,○) or IF2αΔGTPase (▪,□); (**B**) growth of *E. coli* cells expressing IF2α (upper half of the plates) or IF2αΔGTPase (lower half of the plates) for the indicated times at the indicated temperatures on LB agar plates containing Amp (60 μg/ml) and Kan (25 μg/ml). The plates are divided into upper and lower halves by a horizontal line.

At 37°C the cells expressing exclusively the IF2ΔN mutant are viable and do not have any noticeable phenotype, provided that the medium contains >0.01% arabinose which is necessary to activate the P_BAD_ promoter (Figure 3A); only when the arabinose concentration is 0.01% or less, as in the case in which the factor is expressed from the leaky P_BAD_ promoter, the generation times of the cells expressing the deletion mutant become 67 or 90 min, somewhat longer compared to those of the wt cells whose generation time is 45 or 66 min, respectively (Supplementary Figure S7). Also the cells expressing the IF2αΔGTPase mutant are viable at 37°C (26) and display only a slightly reduced growth rate compared to the isogenic wt cells (Figure 4A).

By contrast, when the temperature is lowered to 10°or 15°C the cells expressing IF2ΔN (Figure 3A) and IF2αΔGTPase (Figure 4A) grow very poorly after a long lag or not at all. Furthermore, when the cells expressing IF2ΔN are grown overnight at 37°C and then plated on LB agar containing Amp, Kan and arabinose (see figure legend), they form very few colonies at 15°C and only after 81 h of incubation, unlike the cells expressing IF2α (Figure 3B). On the other hand, the cells expressing IF2αΔGTPase do not form colonies, not even after 210 h at 20°C and 15°C, unlike the isogenic cells expressing IF2α (Figure 4B).

Overall, these data indicate that at and below the temperature (20°C) which triggers cold-stress the lack of the NTD or inactivation of the GTPase activity of IF2 becomes critical, conferring a cold-sensitive phenotype even if transcription of *infB* is not diminished. This finding implies that during cold acclimation IF2 performs a role for which the presence of both its NTD and GTPase activity are necessary. It is also noteworthy that at the optimal growth temperature, in the absence of a sufficient amount of the proper inducer, transcription from the leaky P_BAD_ promoter for the cells expressing IF2ΔN is no longer sufficient to ensure an adequate amount of IF2. Since it is known that the NTD confers upon IF2 a high affinity for the 30S subunits (25,29), it seems likely that the absence of this domain makes the cells sensitive to the cellular concentration of the factor, thereby determining sub-optimal growth conditions.

### Defective assembly/maturation of ribosomal subunits in cold-sensitive IF2 mutants

It is well established that a cold-sensitive phenotype is often associated with a defect in ribosome assembly (37,38). In light of the increased number of IF2 molecules associated with ribosomes during cold acclimation together with several proteins involved in ribosomal subunit assembly and maturation, the following experiments were carried out to investigate if the cold-sensitive phenotype of the cells expressing the two IF2 mutants could be due to a failure of the cell to assemble mature ribosomal subunits.

**Figure 5.**
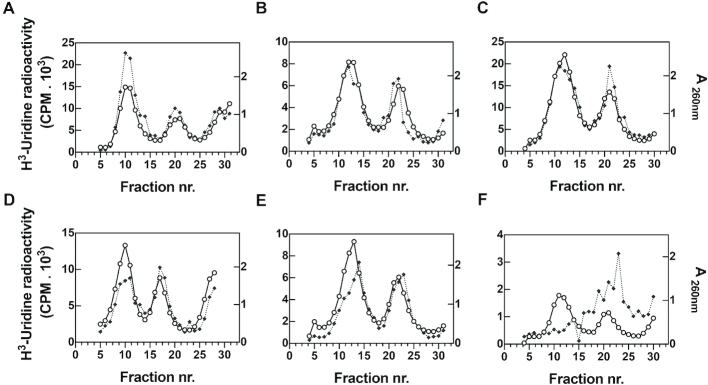
Temperature-dependent effect of IF2 mutations on ribosomal subunit assembly. Sucrose density gradient analysis (sedimentation from right to left) of the ribosomal subunits present in the extracts of cells exposed to [^3^H] uridine as described in Materials and Methods. Extracts of *E. coli* cells expressing IF2α (**A** and **D**), IF2ΔN (**B** and **E**) and IF2αΔGTPase (**C** and **F**) at 37°C (A–C) or 10°C (D–F). The A_260_ (○ solid line) and the [^3^H] uridine incorporated (✦ dotted line) were determined for each fraction.

Sucrose density gradient analysis of the extracts of cells expressing wt and mutant IF2 molecules pulsed with radioactive uridine revealed that neither the IF2αΔGTPase nor IF2ΔN mutation affected ribosomal subunit assembly at 37°C since in all cases the profiles of the radioactive uridine incorporated coincided with the optical density peaks of the 50S and 30S subunits in the extracts of control and mutant cells (Figure 5A–C). However, the situation was quite different when the extracts of cells exposed to 10°C were analyzed. In fact, as judged from the profile of the radioactive uridine incorporated, in cells expressing wt IF2 the assembly of the 50S ribosomal subunits appears slightly retarded compared to the situation observed at 37°C (Figure 5D), whereas more or less severe assembly/maturation defects were detected in the cells expressing the IF2 mutants. In cells expressing IF2ΔN the profile of the incorporated radioactive uridine appears somewhat retarded and coincides only in part with the optical density profile of the two subunits, suggesting the presence of several assembly intermediates of both subunits but mainly of the 50S (Figure 5E).

The assembly defect detected in the cells expressing the IF2αΔGTPase is much more severe. In this case there is no radioactivity associated with the 50S peak and the bulk of radioactivity sediments between the 50S and 30S peaks and slower than the 30S (Figure 5F), indicating severe impairment of the assembly of both ribosomal subunits at 10°C.

### IF2 has protein chaperone activity

The evidence of an involvement of IF2 in the assembly and/or maturation of ribosomal subunits raises questions concerning the mechanism by which the factor could perform this role. A previous study had suggested that IF2 as well as elongation factor EF-G, another ribosome-dependent GTPase, might be endowed with chaperone activity (39). Thus, a possible protein chaperone activity of IF2α and of its mutants was investigated using acid-denatured or heat-denatured green fluorescent protein (GFPS30R).

Experiments were carried out to determine the conditions under which there is a linear relationship between GFP concentrations and fluorescence emission. Because this linearity was found to exist between 1.5 and at least 12 pmol of GFP (Supplementary Figure S2), all experiments were carried out using 7.5 pmol of total GFP, corresponding to 0.075 μM. Other experiments were carried out to determine if proteins other than IF2 and EF-G can promote the refolding of denatured GFP. Aside from bovine serum albumin (BSA), whose protein chaperone activity is well documented (40,41), none of the other proteins tested (i.e. soybean trypsin inhibitor and carbonic anhydrase) had any effect on refolding of denatured GFP.

**Figure 6.**
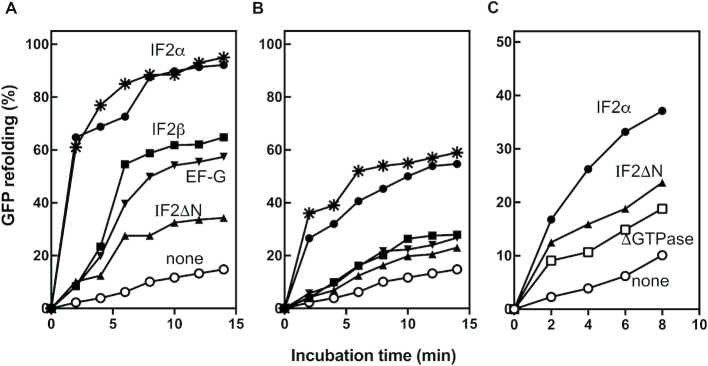
Chaperone-assisted refolding of acid-denatured GFP. Time-dependent refolding at 17°C of acid-denatured GFP in the presence of 1mM GTP and of IF2α (•); IF2β (▪); EF-G (▾) and IF2ΔN (▴) at (**A**) chaperone/GFP molar ratios = 20:1 and at (**B**) chaperone/GFP ratios = 1:1; renaturation in the presence of IF2α was carried out also at 25°C (*); (**C**) comparison of the chaperone activity of IF2α (•); IF2ΔN (▴) and IF2αΔGTPase (□) at chaperone/GFP molar ratios = 1:1 as a function of the indicated times of incubation. The curves describing the spontaneous GFP refolding in the absence of chaperones are indicated (○) in all three panels. IF2β is a naturally occurring shorter form of IF2 lacking 158 N-terminal residues (29).

A direct comparison of the time-course of GFP refolding in the presence of IF2α, IF2β, IF2ΔN and EF-G at a stoichiometric ratio of 20:1 (Figure 6A) or 1:1 (Figure 6B) with respect to denatured GFP shows that only IF2α can promote the almost complete (>90%, as judged from the increase of the fluorescence emission) time-dependent refolding of GFP, being substantially more active than either IF2β or EF-G whereas very little refolding occurs in the presence of IF2ΔN. Furthermore, IF2α displays chaperone activity below (i.e. at 17°C, the lowest temperature at which for technical reasons the refolding can be carried out) and above (25°C) the cold stress threshold temperature (Figure 6A, B). The IF2αΔGTPase appears to be even more severely impaired than IF2ΔN in promoting the refolding of denatured GFP (Figure 6C). Similar results were obtained when the rate of refolding of acid-denatured GFP was monitored as a function of increasing concentrations of IF2α (Supplementary Figure S8A), IF2β (Supplementary Figure S8B), IF2ΔN (Supplementary Figure S8C) and EF-G (Supplementary Figure S8D). Taken together, these findings are compatible with a cause-effect relationship between the observed defect in ribosome assembly or maturation and the reduced chaperone activity of these mutants.

### Effect of guanine nucleotides on the chaperone activity of IF2

**Figure 7.**
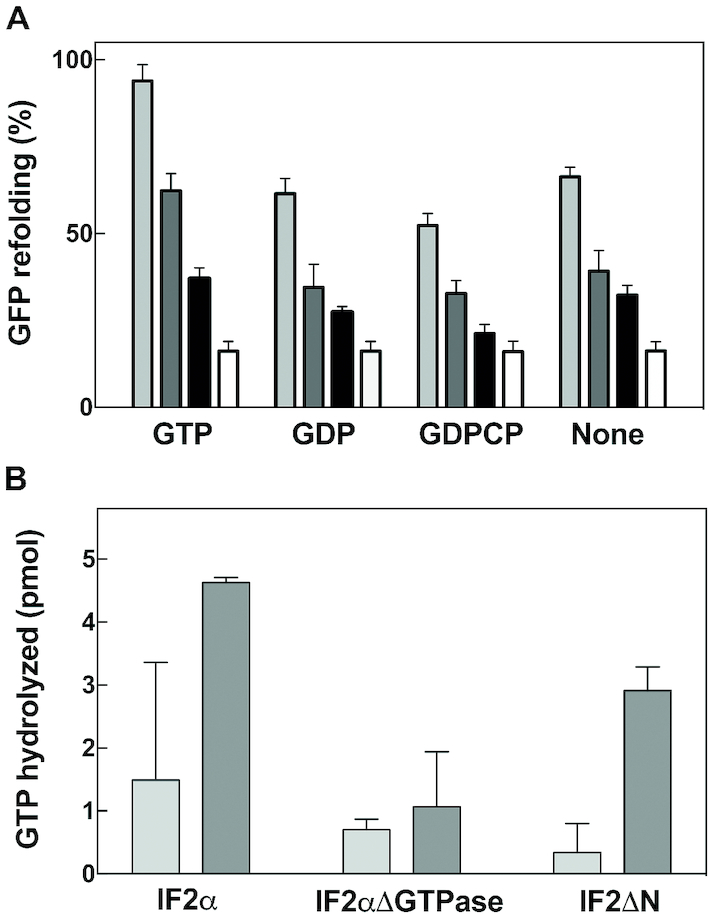
Effect of guanine nucleotides on the chaperone activity of IF2. (**A**) Extent of refolding of acid-denatured GFP after 15 min incubation at 25°C in the presence of IF2α (light gray), IF2β (dark gray), IF2ΔN (black) and no IF2 (white) at chaperone/GFP ratio = 20:1 in the presence of 1 mM of GTP, GDP, GDPCP or in the absence of guanine nucleotides as indicated in the abscissa. (**B**) Amount of GTP hydrolyzed during chaperone-assisted refolding of acid-denatured GFP at chaperone/GFP ratio = 1:1. Level of GTP hydrolysis determined by measuring the amount of Pi produced in the reaction essentially as described (42) in the presence of non-denatured GFP (light grey bars) and acid-denatured GFP (dark grey bars) upon incubation for 8 min at 25°C with IF2α, IF2αΔGTP*ase* and IF2ΔN as indicated in the abscissa.

Because IF2 is a guanine nucleotide binding protein as well as a ribosome-dependent (42) and ribosome-independent (43) GTPase, a possible effect of GTP binding and/or hydrolysis on the chaperone activity of IF2 was investigated. For this purpose, refolding of denatured GFP promoted by IF2α, IF2β, and IF2ΔN was tested in the presence of GTP, GDP, GDPCP and in the absence of any guanosine nucleotide. An effect of GTP on the activity of IF2αΔGTPase was not considered in these experiments in light of the total inactivity of this mutant in GTP hydrolysis (26). As seen in Figure 7A, IF2α displays the highest chaperone activity in the presence of GTP, and its activity is substantially reduced, albeit not completely abolished, in the presence of GDP, or of the non-hydrolysable analogue GDPCP or in the absence of guanine nucleotides. Also the chaperone activity of IF2β and IF2ΔN, although as expected much lower than that of IF2α, displays similar sensitivity to the type of guanine nucleotide present (Figure 7A).

IF2 is known to assume different conformations depending upon the guanine nucleotide ligand bound (44,45); for this reason it seemed important to determine whether the higher level of chaperone activity of IF2 in the presence of GTP stems from the particular conformation that the factor assumes in the presence of this ligand or is accompanied by GTP hydrolysis. As seen in Figure 7B, substantial GTP hydrolysis occurs during the chaperone activity of IF2α, whereas incubation of denatured GFP with either the IF2αΔGTPase or the IF2ΔN mutant results in a much lower level of hydrolysis.

### Chaperone activity of IF2 in the presence of HSP70 proteins

**Figure 8.**
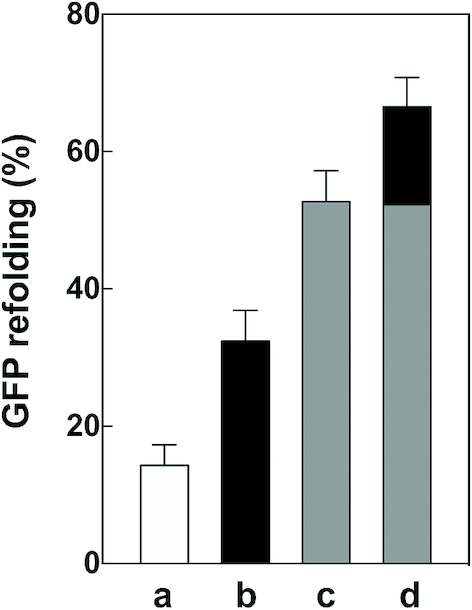
Chaperone activity of IF2 and HSP70 proteins. Refolding of heat-denatured GFP upon incubation for 8 min at 25°C at chaperone/GFP stoichiometric ratios of 1:1 carried out in Buffer containing 1 mM GTP and 1 mM ATP. GFP refolding without chaperone (**a**) and in the presence of IF2α (**b**); DnaK, DnaJ and GrpE (**c**); DnaK, DnaJ, GrpE and IF2α (**d**) in which the grey part of the bar represents the refolding due to the HSP70 chaperone proteins and the black part represents the increased level of refolding due to the presence of IF2α.

The following experiments were carried out to compare the chaperone activity of IF2α alone and in the presence of a mixture of known chaperones of the HSP70 family such as GrpE, DnaJ and DnaK. Heat-denatured, instead of acid-denatured GFP, was used for these experiments in which GFP was incubated at a 1:1 stoichiometric ratio with each of these chaperones which normally deal with heat-shock-denatured proteins. As expected, a mixture of the aforementioned chaperones was found to produce a more extensive renaturation of GFP (∼55%) compared to that obtained in the presence of IF2α alone (∼35%). However, the inclusion of IF2α in the incubation mixture containing the mixture of the HSP70 proteins resulted in a clear increase of the level of renatured GFP which reached >66% of the total protein (Figure 8), indicating that the protein refolding activity of IF2α can be additive with respect to other known chaperones. Furthermore, these results indicate that the chaperone activity of IF2 is not restricted to the refolding of acid-denatured GFP but occurs also with the heat-denatured protein, therefore ruling out the possibility that refolding of acid-denatured GFP by IF2 might be due to a buffering activity of this protein.

## DISCUSSION

Under optimal growth conditions, the IF/ribosome ratio remains constant and translation initiation involves the binding of a single molecule of each initiation factor to the native (i.e. amenable to initiate mRNA translation) 30S subunit, whereas elongating ribosomes are devoid of initiation factors. However, as seen in Results, after cold stress the level of the three factors increases by approximately the same extent (i.e.∼ 3-fold) with respect to the ribosomes and whereas cold-stress-induced IF3 and IF1 were found to play an important role in ensuring translation of cold-shock transcripts at low temperature, no similar role could be detected for IF2 (3).

The present data indicate instead that the extra copies of IF2 might be essential to ensure successful assembly and/or maturation of the ribosomal subunits during cold acclimation, probably as a result of their protein chaperone activity demonstrated in this study. In fact, we have shown that IF2α can elicit the time-dependent, concentration-dependent refolding of acid- or heat-denatured GFP. In comparison, the shorter naturally occurring form of the factor (IF2β) displays a less efficient chaperone activity. Because the activity of IF2 (α and β) are indistinguishable in all partial reactions of the translation initiation pathway, to the best of our knowledge their different efficiency in chaperone activity represents the first case in which a functional difference between these two forms has been detected.

Compared to IF2α, two IF2 mutants, one bearing a single amino acid substitution (E571K) in the IF2-G3 domain which completely inactivates the GTPase activity without impairing the binding of guanine nucleotides (26) and the other a deletion mutant lacking the entire NTD (294 residues) (29), display strongly impaired chaperone activity. The *E. coli* strains bearing these IF2 mutations have no or only marginal phenotypes at 37°C, but are cold-sensitive, being unable to grow at the temperatures which elicit the cold-shock response and display a defect in ribosomal subunits assembly and/or maturation. Indeed, the sucrose density gradient analyses of the extracts of these cold sensitive mutants suggest that they accumulate immature precursors of both ribosomal subunits. In this connection, it should be noticed that although the extra molecules of IF2 synthesized upon cold stress are found in association with a ‘30S subunits peak’, this does not imply that IF2 is exclusively involved with assembly/maturation of the small subunit. In fact, the ‘30S peak’ pooled for MS analysis is heterogeneous and likely comprises precursors of both subunits, as indicated by the fact that it contains almost the same amount of S-proteins and L-proteins (Supplementary Figure S9).

Despite their different natures, both IF2 mutations cause a defect in the assembly and/or maturation and it seems relevant to notice that a strongly reduced protein chaperone activity is the property which the two mutants have in common and that the phenotype of cells expressing IF2αΔGTPase is more severe in both chaperone activity and ribosomal subunit maturation. Although a more severe defect in subunit assembly may possibly reflect a defect in an earlier step of the process, it is tempting to suggest the existence of a cause-effect relationship between the defect in chaperone activity and ribosome assembly/maturation.

Also consistent with the premise that IF2 plays a role in ribosome assembly and/or maturation is the association of the extra copies of IF2 with the 30S peak along with extra copies of several other proteins such as CsdA (46), RbfA (47,48), PNPase (49), RNaseR (23) SrmB and LepA (50), YibL (51,52) MraW (RsmH) (23,53) and KsgA (54) which have been implicated in ribosome assembly and in mRNA and rRNA quality control and degradation during cold-adaptation (55). Furthermore, it is noteworthy that cold stress induces the expression of the entire *nusA-infB* operon (24) which, in addition to IF2, encodes other proteins (RimP, NusA, RbfA) also implicated in ribosome assembly or maturation (56–58). It seems highly unlikely that all these occurrences are coincidental; instead they seem to be converging clues which confirm that IF2 plays a role in ribosome assembly/maturation.

It is interesting to note that, in addition to the two IF2 mutations described here, there are also other IF2 mutations which have been shown to be cold-sensitive; in one case, a V791I substitution in the C2 domain of the factor (see Figure 1 of (59)) was found to affect the cold stress adaptation of *E. coli*. In this case, the cold sensitivity was likely due to a defect in the interaction between the initiation factor and ribosomal protein S12 which may have no bearing on the ribosomal subunit assembly/maturation (59). Cold sensitive phenotypes were also reported for IF2 mutations acting as extragenic suppressors of the *E. coli* protein export gene *secY* as well as of other secretion-defective mutations (60,61).

Finally, our data indicating an involvement of IF2 in ribosomal subunit assembly and maturation is in full agreement with the finding that lamotrigine, a small molecule which binds to IF2 without inhibiting translation, causes cold sensitivity accompanied by a rapid accumulation of immature 30S and 50S subunits and that suppressors of lamotrigine activity were found to map within the *infB* gene (62).

In conclusion, the present data as well as published results mentioned above provide a strong indication that translation initiation factor IF2 is yet another factor which should be added to the long list of proteins (50), many of them endowed with GTPase activity (63,64), which take part in the assembly and maturation of the ribosomal subunits. Our results further indicate that this activity, for which the cell requires an increased number of IF2 molecules, is essential during the cold acclimation phase which follows cold stress when this process becomes particularly critical.

## Supplementary Material

Supplementary Data

## Notes

Present address: Anna Brandi, School of Biosciences and Veterinary Medicine, University of Camerino.

Present address: Lolita Piersimoni, Biological Chemistry, Medical School, University of Michigan, Ann Arbor, MI 48109, USA.

Present address: Naser Aliye Feto, OMICS Research Group & Facility: Department of Biotechnology, Faculty of Applied & Computer Sciences, Vaal University of Technology, Vanderbijlpark, South Africa.

Present address: Jean-Hervé Alix, CNRS UMR 8261. Institut de Biologie Physico-Chimique, Université Paris 7-Denis Diderot, Paris, France.

Present address: Frank Schmidt, Proteomics Core, Weill Cornell Medicine-Qatar, Qatar Foundation - Education City, PO box 24144 Doha, State of Qatar.
