# Supplementary material for: Translation initiation factor IF2 contributes to ribosome assembly and maturation during cold adaptation
Source: Nucleic Acids Res. 2019 Mar 27;47(9):4652–62. doi: 10.1093/nar/gkz188 (PMC6511846; doi:10.1093/nar/gkz188)
Supplement: Supplementary Data [file gkz188_supplemental_files.pdf]

SUPPLEMENTARY DATA

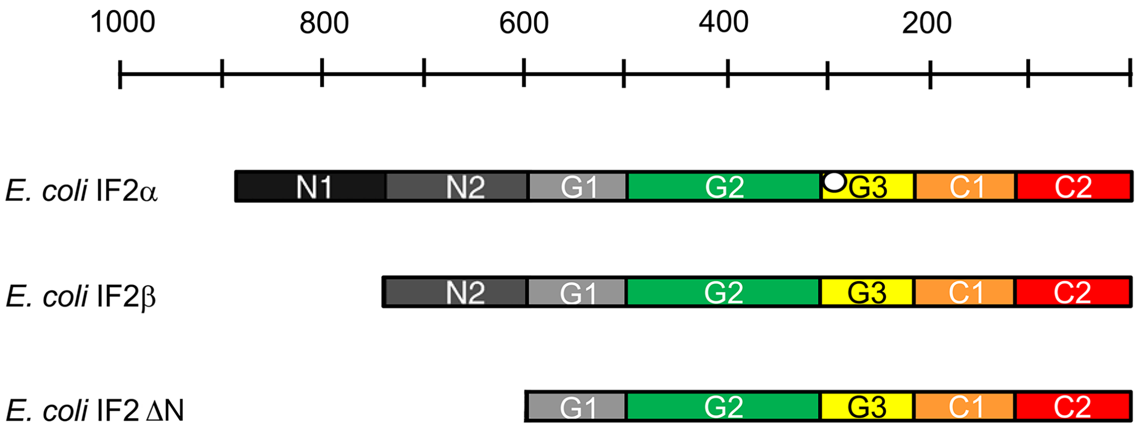

Figure S1 Schematic representation of the domain structure of the two naturally occurring forms of *E. coli* IF2, namely IF2α and IF2β as well as of the IF2ΔN mutant. The position of the single amino acid substitution (E571K) which inactivates the GTPase activity of the factor is shown by a white dot within domain G3 of the factor

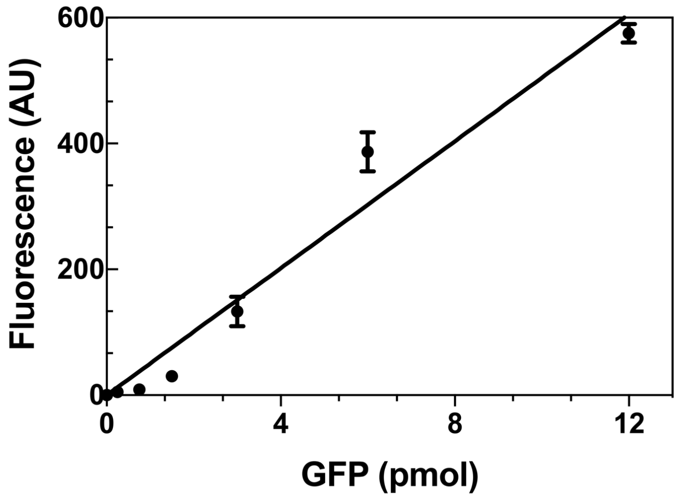

Figure S2 Linear relationship between amount of GFP and fluorescence emission.

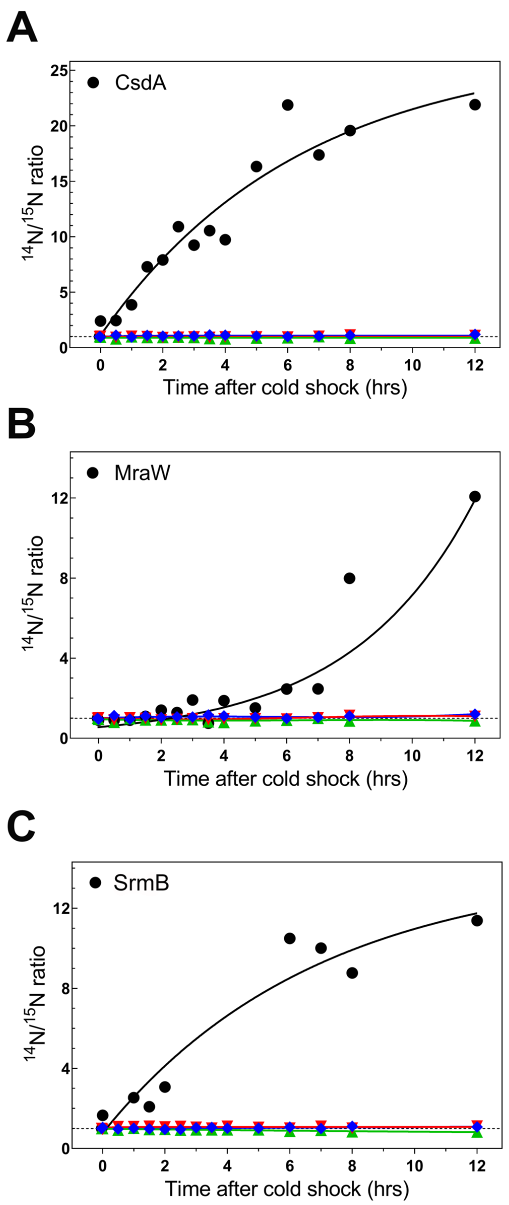

Figure S3. (A) Variation, as a function of the time elapsed after cold stress (37°C →10°C), of the $^{14}N/^{15}N$ ratios for proteins associated with 30S subunits (A) CsdA (●) and ribosomalproteins S12 (▲), S13 (▼) and S14 (♦); (B) MraW (●) and ribosomal proteins S12 (▲), S13 (▼) and S14 (♦) and (C) SrmB (●) and ribosomal proteins S2 (▲), S3 (▼) and S4 (♦). The ratios recorded just before the stress are = 1.

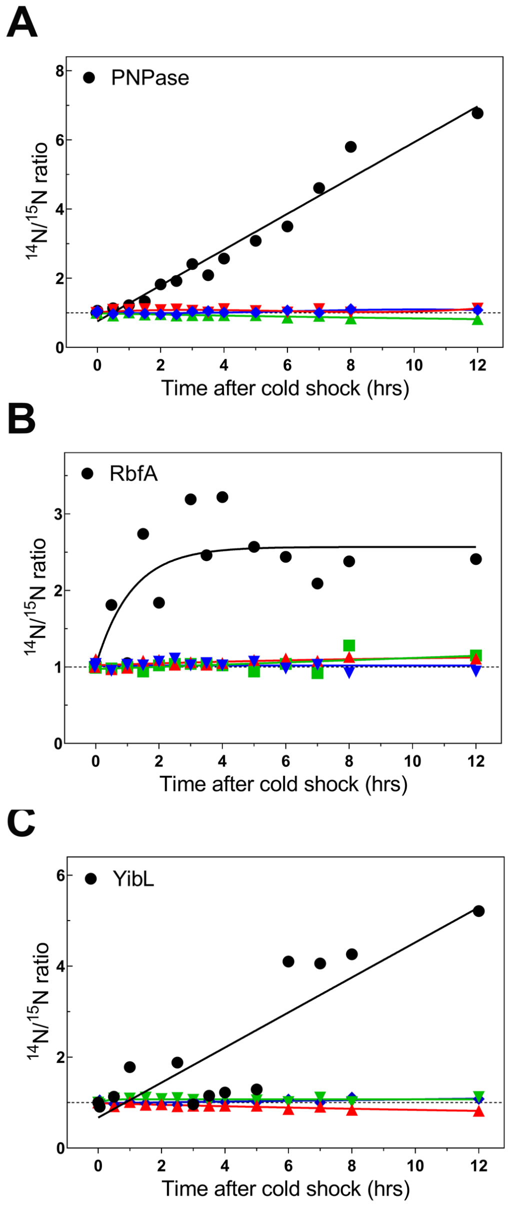

Figure S4 Variation, as a function of the time elapsed after cold stress (37°C →10°C), of the $^{14}N/^{15}N$ ratios for proteins associated with 30S subunits (A) PNPase (●) and ribosomal proteins S2 (▲), S3 (▼) and S4 (♦); (B) RbfA (●) and ribosomal proteins S5 (▲), S6 (▼) and S7 (♦) and (C) YibL (●) and ribosomal proteins S2 (▲), S3 (▼) and S4 (♦). The ratios recorded just before the stress are = 1.

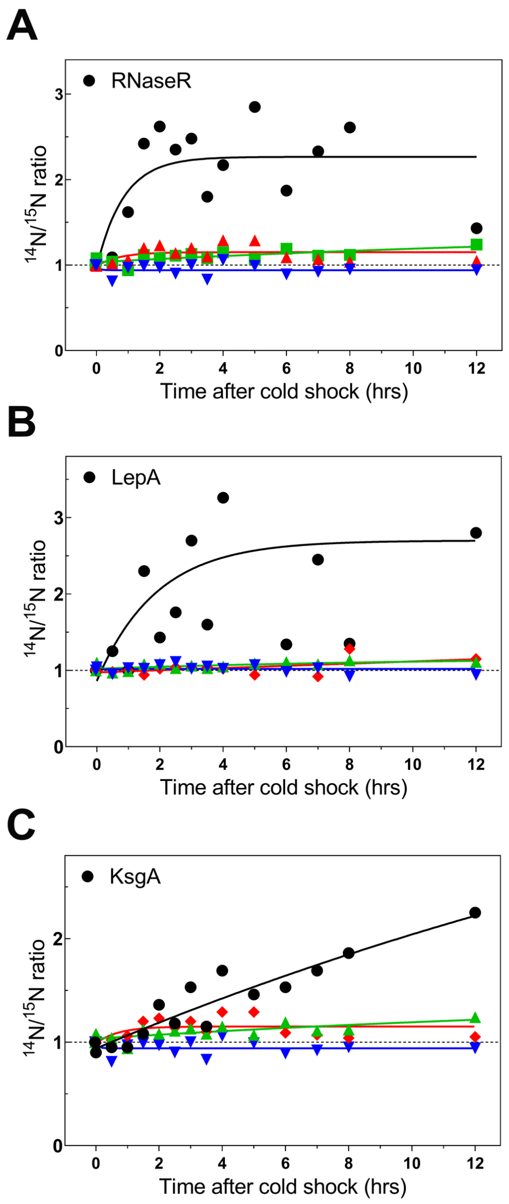

Figure S5 Variation, as a function of the time elapsed after cold stress (37°C →10°C), of the $^{14}N/^{15}N$ ratios for proteins associated with 30S subunits (A) RNaseR (●) and ribosomal proteins S8 (■), S9 (▲) and S10 (▼) and (B) LepA (●) and ribosomal proteins S5 (■), S6 (▲) and S7 (▼) and (C) KsgA (●) and ribosomal proteins S8 (▲), S9 (♦) and S10 (▼). The ratios recorded just before the stress are = 1.

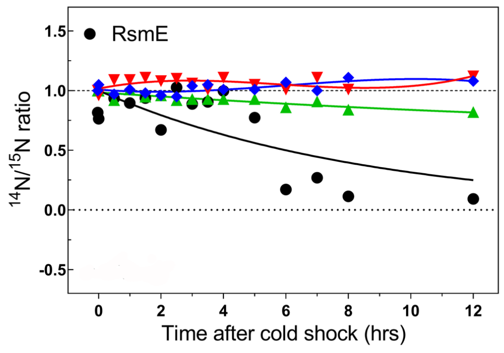

Figure S6 Variation, as a function of the time elapsed after cold stress (37°C →10°C), of the $^{14}N/^{15}N$ ratios for RsmE protein (●) and for ribosomal proteins S4 (♦), S3(▼), S2 (▲) associated with 30S subunits. The ratios recorded just before the stress are = 1.

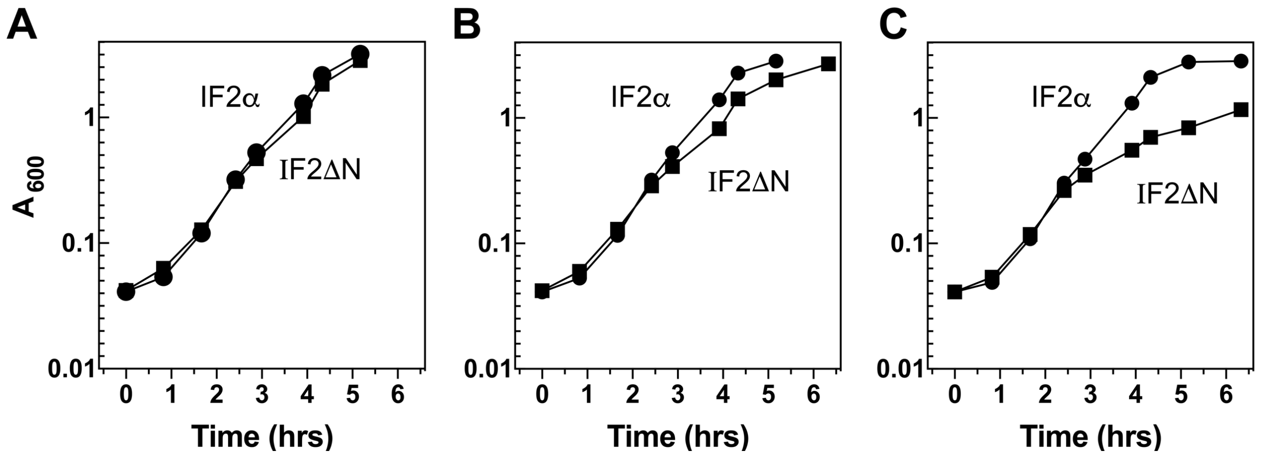

Figure S7 Growth curve at 37°C of *E. coli* IF2α (●) and *E. coli* IF2ΔN (■) in LB medium containing Amp (60µg/ml), Kan (25µg/ml) and 0,025% (A), 0.01% (B) or without arabinose (C).

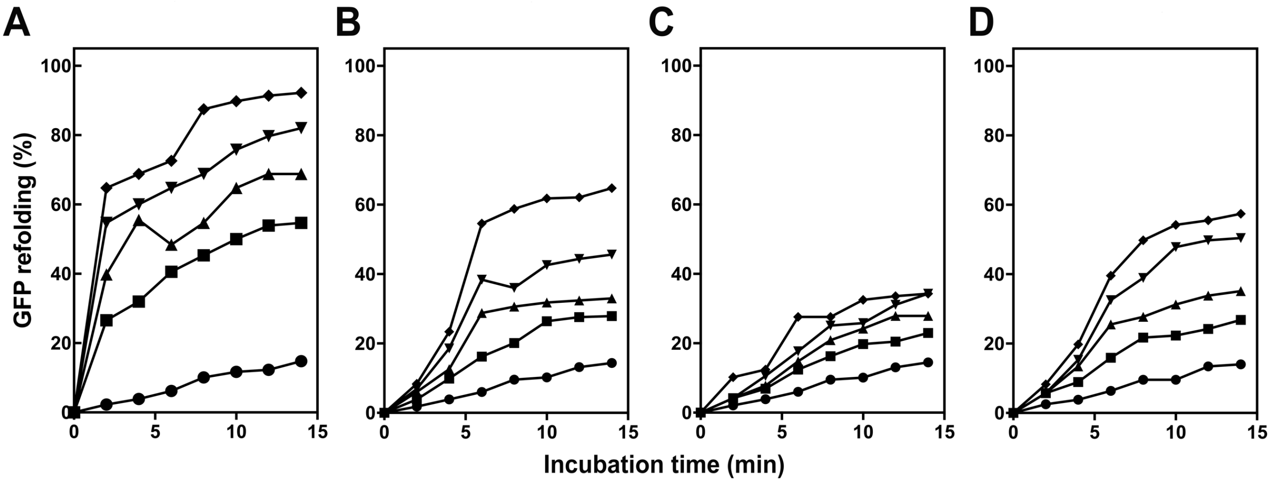

Figure S8 Time-dependent refolding at 25°C of acid-denatured GFP in the presence of 1mM GTP and of increasing concentrations of (A) IF2α; (B) IF2β; (C) IF2ΔN and (D) EF-G at stoichiometric chaperone/GFP ratios = ■ 1:1; ▲5:1; ▼10:1; ◆ 20:1. Spontaneous refolding of GFP in the absence of chaperone (●).

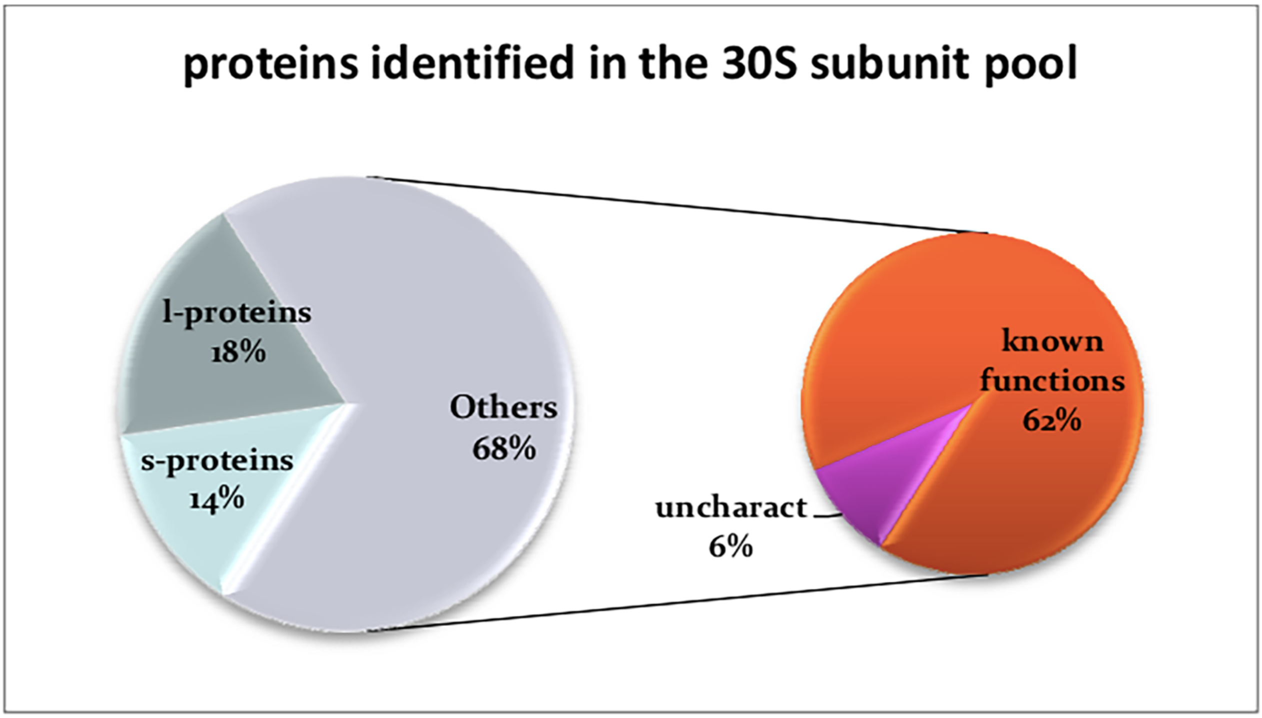

Figure S9 Nature of the proteins identified by mass spectrometry in association with the pool of 30S ribosomal subunits resolved by centrifugation on sucrose density gradients.
